# Fatigue, sexual function and mood following treatment for haematological malignancy: the impact of mild Leydig cell dysfunction

**DOI:** 10.1054/bjoc.1999.1000

**Published:** 2000-02

**Authors:** S J Howell, J A Radford, E M A Smets, S M Shalet

**Affiliations:** Departments of Endocrinology and Medical Oncology, Christie Hospital NHS Trust, Withington, Manchester, M20 4BX, UK; Department of Medical Psychology, Academic Medical Centre, University of Amsterdam, PO Box 22700, Amsterdam, DE, 1100, The Netherlands

**Keywords:** Leydig cell, testosterone, fatigue, sexual, mood

## Abstract

Fatigue, sexual dysfunction, anxiety and depression are all more common in patients who have previously been treated with cytotoxic chemotherapy and radiotherapy (XRT) for haematological malignancies. Following therapy, a significant proportion of men have biochemical evidence of Leydig cell dysfunction, defined by a raised luteinizing hormone level in the presence of a low/normal testosterone level. We postulated that mild testosterone deficiency may account for some of the long-term side-effects of treatment, and we have therefore assessed fatigue, mood and sexual function by questionnaire in 36 patients with Leydig cell dysfunction (group 1), and also in a group of 30 patients (group 2) with normal hormone levels who underwent the same treatment for cancer. There was no significant difference in anxiety and depression scores between the two groups although anxiety scores were higher than those previously reported for normal men. Eighty-seven per cent of group 2 were sexually active compared with only 69% of group 1 (*P* = 0.1), and patients in group 1 engaged less in sexual activity than those in group 2 (mean of 1.8 times per week compared with 3.2 times per week;*P* = 0.02) Fatigue scores were significantly higher in both groups compared with normal men, but there were no significant differences in any of the fatigue subscales between the two groups. We conclude that mild Leydig cell insufficiency following treatment with cytotoxic chemotherapy ± XRT is not associated with higher levels of fatigue and anxiety but may result in reduced sexual function. These results do not provide a convincing argument that androgen replacement therapy is mandatory to improve quality of life in the majority of these patients, although it may be beneficial in a minority. To establish criteria for selection of patients for a trial of androgen therapy a randomized placebo-controlled study will be necessary. © 2000 Cancer Research Campaign


*British Journal of Cancer* (2000) **82**(4), 789–793
© 2000 Cancer Research Campaign
DOI: 10.1054/ bjoc.1999.1000, available online at http://www.idealibrary.com on IDEAL®

### Fatigue, sexual function and mood following treatment for haematological malignancy: the impact of mild Leydig cell dysfunction

**SJ Howell[1], JA Radford[2], EMA Smets[3] and SM Shalet[1]**

Departments of [1]Endocrinology and [2]Medical Oncology, Christie Hospital NHS Trust, Withington, Manchester M20 4BX, UK; [3]Department of Medical Psychology, Academic Medical Centre, University of Amsterdam, PO Box 22700, 1100 DE Amsterdam, The Netherlands

**Summary** Fatigue, sexual dysfunction, anxiety and depression are all more common in patients who have previously been treated with cytotoxic chemotherapy and radiotherapy (XRT) for haematological malignancies. Following therapy, a significant proportion of men have biochemical evidence of Leydig cell dysfunction, defined by a raised luteinizing hormone level in the presence of a low/normal testosterone level. We postulated that mild testosterone deficiency may account for some of the long-term side-effects of treatment, and we have therefore assessed fatigue, mood and sexual function by questionnaire in 36 patients with Leydig cell dysfunction (group 1), and also in a group of 30 patients (group 2) with normal hormone levels who underwent the same treatment for cancer. There was no significant difference in anxiety and depression scores between the two groups although anxiety scores were higher than those previously reported for normal men. Eighty-seven per cent of group 2 were sexually active compared with only 69% of group 1 ($P = 0.1$), and patients in group 1 engaged less in sexual activity than those in group 2 (mean of 1.8 times per week compared with 3.2 times per week; $P = 0.02$) Fatigue scores were significantly higher in both groups compared with normal men, but there were no significant differences in any of the fatigue subscales between the two groups. We conclude that mild Leydig cell insufficiency following treatment with cytotoxic chemotherapy ± XRT is not associated with higher levels of fatigue and anxiety but may result in reduced sexual function. These results do not provide a convincing argument that androgen replacement therapy is mandatory to improve quality of life in the majority of these patients, although it may be beneficial in a minority. To establish criteria for selection of patients for a trial of androgen therapy a randomized placebo-controlled study will be necessary. © 2000 Cancer Research Campaign

**Keywords:** Leydig cell; testosterone; fatigue; sexual; mood

Improving survival remains the primary objective of therapy for most treatable cancers. However, as rates of long-term remission and cure following therapy for certain malignancies improve, achieving this with a minimum of late side-effects is becoming more important. There is a great deal of evidence to suggest that various aspects of quality of life are reduced in survivors of cancer (Fobair et al, 1986; Berglund et al, 1991; Bjordal et al, 1994; Hjermstad and Kaasa, 1995; Greenberg et al, 1997). Therefore, assessment of quality of life after therapy is a vital part of determining the overall efficacy of different treatment regimens. Furthermore, it is important to delineate a cause for these side-effects as this may facilitate the development of strategies for their prevention or treatment.

Alterations in mood are very common in long-term survivors of malignancy. An increased incidence of anxiety and depression has been described after treatment of various cancers (Fobair et al, 1986; Devlen et al, 1987; Ahles et al, 1996; Aass et al, 1997) using various different standardized questionnaires. In addition, sexual dysfunction is a relatively frequent problem in patients following treatment for malignancy (Fobair et al, 1986; Bloom et al, 1993; Hjermstad and Kaasa, 1995), although the incidence varies according to the type of malignancy and treatment received.

Fatigue is the most commonly reported symptom in cancer patients. During chemotherapy or radiotherapy fatigue is experienced by the vast majority of patients (Smets et al, 1993) and long-term follow-up has suggested that in some patient groups this may persist for many years. However, most studies in cancer patients have assessed fatigue by single items in a general symptom checklist (Smets et al, 1993) and therefore do not provide an accurate estimation of the degree of fatigue experienced by these patients.

Androgen deficiency is associated with increased fatigue, sexual dysfunction and alterations in mood (Burris et al, 1992), and testosterone replacement has been shown to improve these abnormalities (Arver et al, 1996; Wang et al, 1996). We have demonstrated that approximately one-third of men treated with gonadotoxic chemotherapy for various haematological malignancies have biochemical evidence of Leydig cell dysfunction (Howell et al, 1999), and we have therefore hypothesized that mild androgen deficiency may account for the increase in fatigue, sexual dysfunction and alterations in mood observed in a proportion of the patients treated with cytotoxic chemotherapy and radiotherapy for cancer. In order to assess the contribution of mild testosterone deficiency to alterations in these parameters we have assessed energy, sexual function and mood in a group of men with Leydig cell dysfunction following successful cancer treatment and compared the results with a similar cohort who had entirely

*Received 6 May 1999*
*Revised 11 September 1999*
*Accepted 24 September 1999*

*Correspondence to:* SM Shalet

**Table 1** Diagnosis and chemotherapy regimens used for treatment of the patients and controls

| | Diagnosis | | Treatment | |
|---|---|---|---|---|
| Group 1 | Hodgkin's disease | 25 | MVPP alone | 12 |
| | | | Hybrid alone | 6 |
| | | | MVPP + Hybrid | 3 |
| | | | HDCT[a] | 4 |
| | Non-Hodgkin's lymphoma | 4 | HDCT + VAPEC-B | 3 |
| | | | HDCT + CHOP | 1 |
| | Multiple myeloma | 4 | HDCT | 4 |
| | Acute myeloid leukaemia | 2 | HDCT | 2 |
| | Acute lymphoblastic leukaemia | 1 | HDCT | 1 |
| Group 2 | Hodgkin's disease | 22 | MVPP alone | 4 |
| | | | Hybrid alone | 13 |
| | | | MVPP + Hybrid | 2 |
| | | | HDCT[a] | 3 |
| | Non-Hodgkin's lymphoma | 3 | HDCT + VAPEC-B | 3 |
| | Acute myeloid leukaemia | 5 | HDCT | 5 |

MVPP – Mustine, vinblastine, procarbazine and prednisolone. Ch1VPP/EVA Hybrid – chlorambucil, vinblastine, procarbazine, prednisolone, etoposide, vincristine and doxorubicin. HDCT – High-dose chemotherapy. VAPEC-B – Vincristine, doxorubicin, prednisolone, etoposide, cyclophosphamide and bleomycin. CHOP – cyclophosphamide, doxorubicin, vincristine and prednisolone. [a]HDCT for relapse following treatment with Ch1VPP/EVA Hybrid, MVPP or both.

normal hormone levels. In addition, we have also compared the results to those obtained in healthy men, where such data are available, so that we can also assess the overall impact of the malignancy and its treatment.

### PATIENTS

We screened a total of 209 men who had been treated with either MVPP (mustine, vinblastine, procarbazine and prednisolone), Ch1VPP/EVA hybrid (chlorambucil, vinblastine, procarbazine, prednisolone, etoposide, vincristine and doxorubicin) or high-dose chemotherapy (either cyclophosphamide, BCNU and etoposide, busulphan and cyclophosphamide, high-dose cyclophosphamide or high-dose melphalan). All patients were in remission at the time of analysis, and had completed chemotherapy at least 12 months previously. None had any history of testicular disease or had received radiotherapy to the testes. Leydig cell impairment was defined arbitrarily by a raised luteinizing hormone (LH) level ($\geq 8$ IU $l^{-1}$) in the presence of a testosterone level in the lower half of the normal range or frankly subnormal ($< 20$ mmol $l^{-1}$) (Howell et al, 1999). We identified a total of 67 men who satisfied these criteria for impaired Leydig cell function, and of these, 36 men completed the questionnaires (group 1). Of the remaining 142 patients, 95 had entirely normal hormone levels (testosterone $> 10$ nmol $l^{-1}$ and LH $< 8$ IU $l^{-1}$), and of these, 61 men were approached to complete the questionnaires. These patients were selected on the basis of hormone levels, and in addition an attempt was made to match this group for age with the 36 patients with Leydig cell impairment. Of the 61 men approached, 30 completed the questionnaires (group 2). There was no significant difference in diagnosis, treatment, or hormone levels between those subjects who completed the questionnaires and those who did not, and the 66 patients in the study are therefore representative of the cohort as a whole. The diagnosis and treatment of these 66 patients is shown in Table 1. In addition to chemotherapy, 39 men received radiotherapy (XRT): 25 of the 36 patients and 15 of the controls. The neck, chest and mantle areas were the areas irradiated in the majority of subjects, although seven received total body irradiation before bone marrow transplantation (BMT) and five received XRT to the abdomen or pelvis.

All 66 patients completed three questionnaires. Mood was assessed using the hospital anxiety and depression scale (HADS). This is a 14-item questionnaire which has been well validated as a tool for identifying abnormal mood states. The HADS has been used in studies of distress in cancer patients in general (Carroll et al, 1993; Ibbotson et al, 1994; Aass et al, 1997) and specifically in Hodgkin's disease (Loge et al, 1997). It has been shown to perform well in cancer patients free of disease (Ibbotson et al, 1994). The HADS contains two 7-item scales, one for depression and one for anxiety, both with a score range of 0–21. A score of eight or above suggests the possibility of clinical disease (Zigmond and Snaith, 1983; Carroll et al, 1993; Aass et al, 1997), whilst a score of $\geq 11$ is indicative of likely anxiety or depression (Zigmond and Snaith, 1983). The total score has also been used to determine the presence of abnormal mood states, with cut-offs of 13 and 19 being used previously in studies of cancer patients (Razavi et al, 1990; Carroll et al, 1993).

Sexual function was assessed using a questionnaire adapted from a sexual diary developed for trials of the male contraceptive pill. It asked the subjects about their interest in sex, frequency and enjoyment of intercourse and masturbation, and frequency of early morning erections over the previous 7 days.

Fatigue was assessed using a questionnaire specifically designed for cancer patients; the multidimensional fatigue inventory (MFI) 20. This is a 20-item questionnaire which has been validated in patients with malignant disease (Smets et al, 1996). The MFI-20 assesses five different aspects of fatigue: general fatigue, physical fatigue, reduced activity, reduced motivation and mental fatigue, with a score of 4–20 for each section. Normal data for the MFI-20 from a group of 139 healthy Dutch subjects have previously been reported (Smets et al, 1998). Forty-four of these individuals were males under the age of 60 years, and this cohort was used for comparison with the cancer patients (normals). Information regarding education level was also collected for all the subjects as this has been found to correlate with the incidence of anxiety and depression (Loge et al, 1997).

 *© 2000 Cancer Research Campaign*

**Table 2** Age, number of cycles of chemotherapy, education level, time since treatment and hormonal parameters in the patients and controls. Age, number of cycles and years since treatment are expressed as medians (range), and hormonal variables are expressed as means (standard deviation)

| | Group 1 ($n$ = 36) | Group 2 ($n$ = 30) | Normals ($n$ = 44) |
|---|---|---|---|
| Age (years) | 42 (27–52) | 38.5 (21–51) | 40.3 (20–59) |
| No. of cycles | 7.5 (3–11) | 7.0 (6–11) | – |
| Education level | | | |
| Less than 16 years | 1 | 3 | 2 |
| Up to 16 years | 25 | 14 | 8 |
| Up to 18 years | 6 | 8 | 24 |
| Beyond 18 years | 4 | 5 | 10 |
| Years since treatment | 8.0 (1–21) | 8.0 (1–17) | – |
| Testosterone (nmol $l^{-1}$) | 13.5 (3.5)[a] | 20.2 (6.9) | – |
| SHBG (nmol $l^{-1}$) | 30.6 (13.3) | 31.4 (8.0) | – |
| LH (IU $l^{-1}$) | 10.2 (2.4)[a] | 5.4 (1.5) | – |
| FSH (IU $l^{-1}$) | 23.8 (7.8)[a] | 13.2 (5.9) | – |
| Testosterone/SHBG | 0.51 (0.21)[b] | 0.66 (0.19) | – |
| Testosterone/LH | 1.4 (2.45) | 4.1 (0.48)[a] | – |

[a]$P < 0.0001$; [b]$P = 0.002$.

**Table 3** Mean HAD scores for patients and controls and number (%) with an anxiety or depression score of ≥ 8 and ≥ 11, and number (%) with a total score ≥ 13 and ≥ 19.

| | | Group 1 ($n$ = 36) | Group 2 ($n$ = 30) | All ($n$ = 66) |
|---|---|---|---|---|
| Anxiety | Mean score | 7.4 | 6.5 | 7.0 |
| | ≥ 8 | 14 (39%) | 13 (43%) | 27 (41%) |
| | ≥ 11 | 7 (19%) | 3 (10%) | 10 (15%) |
| Depression | Mean score | 4.1 | 3.7 | 3.9 |
| | ≥ 8 | 5 (14%) | 3 (10%) | 8 (12%) |
| | ≥ 11 | 1 (3%) | 0 | 1 (2%) |
| Total | Mean score | 11.6 | 10.2 | 11 |
| | ≥ 13 | 15 (42%) | 11 (37%) | 26 (39%) |
| | ≥ 19 | 3 (8%) | 1 (3%) | 4 (6%) |

**Table 4** Sexual function in the patients and controls over the previous 7 days. Sexual activity refers to either intercourse or masturbation. Enjoyment and sexual interest were scored by patients between 1 and 5 and results are expressed as mean scores

| | Group 1 ($n$ = 36) | Group 2 ($n$ = 30) |
|---|---|---|
| Sexual activity | | |
| Yes/No | 25/11 (69%/31%) | 26/4 (87%/13%) |
| Frequency (times/week) | 1.8 | 3.2[a] |
| Ejaculatory problems | 0 | 0 |
| Enjoyment score | 4.1 | 4.1 |
| Early morning erections | | |
| Yes/No | 25/11 (69%/31%) | 20/10 (67%/33%) |
| Frequency (times/week) | 1.8 | 2.3 |
| Sexual interest | 3.0 | 3.4 |

[a]$P = 0.02$.

Mann–Whitney *U* and Student's *t*-tests were used to compare non-parametric and parametric data between groups. The influence of independent parameters on quality of life measures was assessed using multivariate regression analysis, whilst correlations between outcome variables were expressed as Pearson's or Spearman's correlation coefficients ($r$).

### RESULTS

Mean age, level of education, mean number of cycles of chemotherapy, time since treatment and hormone levels in group 1, group 2 and normal men are displayed in Table 2. There were no significant differences in age between any of the groups, and no significant differences in number of cycles of chemotherapy, time since treatment or level of education between group 1 and 2. Patients in group 1 had significantly higher LH and follicle stimulating hormone (FSH) levels and significantly lower testosterone levels than group 2 patients.

There were no significant differences in mean anxiety or depression scores between patients in group 1 and group 2, and no significant difference in the proportion of subjects in each of the groups who had scores above the two suggested cut-offs. Forty-one per cent of the whole cohort had an anxiety score greater than seven, with 15% having a score above 10, whilst only 12% and 2% had depression scores above seven and 10 respectively. Anxiety and depression scores were significantly correlated with each other ($r = 0.54$, $P < 0.001$), but not with any of the hormonal parameters. Patients treated with XRT had a significantly higher mean depression score than those who were not (4.4 vs 2.9; $P = 0.03$).

The results concerning sexual function are illustrated in Table 4. Eighty-seven per cent of group 2 were sexually active compared with only 69% of group 1 ($\chi^2$ test $P = 0.1$). In addition, group 1 patients engaged less in sexual activity than those in group 2 (mean of 1.8 times per week compared with 3.2 times per week; $P = 0.02$) and tended to have fewer early morning erections (mean per week 1.8 vs 2.3; $P = 0.4$). No patients reported any problems with ejaculation or enjoyment of intercourse.

Mean fatigue scores for the patients are displayed in Table 5 along with mean scores obtained from normal healthy men. There were no significant differences in any of the fatigue subscales between groups 1 and 2. Compared with normal men the patients had significantly higher mean general fatigue, physical fatigue and mental fatigue. When the 66 subjects were split according to treatment with XRT it was apparent that compared with patients not treated with XRT, those who received XRT had significantly higher mean scores for general fatigue (13.4 vs 11.5; $P < 0.05$),

© 2000 Cancer Research Campaign

**Table 5** Mean fatigue scores for patients, controls and normal healthy men

| | Group 1 (n = 36) | Group 2 (n = 30) | All subjects (n = 66) | Normal men (n = 44) |
|---|---|---|---|---|
| General fatigue | 12.8[b] | 12.6[b] | 12.7[c] | 9.6 |
| Physical fatigue | 12.5[d] | 11.5[b] | 12.0[d] | 8.5 |
| Reduced activity | 9.3 | 10.0 | 9.6 | 8.7 |
| Reduced motivation | 9.0 | 9.3 | 9.1[a] | 8.3 |
| Mental fatigue | 10.6[a] | 10.8[a] | 10.7[b] | 8.2 |

[a]$P < 0.05$, [b]$P < 0.005$, [c]$P < 0.001$, [d] $P < 0.0001$ compared with normal men.

**Table 6** Relationship between scores for mood, sexual function and fatigue

| | Depression | | Anxiety | |
|---|---|---|---|---|
| | Correlation coefficient | P-value | Correlation coefficient | P-value |
| Sexual interest | –0.38 | = 0.001 | | NS |
| Sexual activity | –0.33 | = 0.007 | | NS |
| General fatigue | 0.64 | < 0.001 | 0.47 | < 0.001 |
| Physical fatigue | 0.57 | < 0.001 | | NS |
| Reduced activity | 0.54 | < 0.001 | 0.31 | = 0.01 |
| Reduced motivation | 0.53 | < 0.001 | 0.52 | < 0.001 |
| Mental fatigue | 0.46 | < 0.001 | 0.52 | < 0.001 |

NS = non-significant.

and a non-significantly trend towards higher scores for mental fatigue (11.3 vs 9.2; $P = 0.07$).

Multiple linear regression analysis was performed to assess the relative effects on quality of life of age, hormone levels, diagnosis, chemotherapy regimen, treatment with XRT, number of cycles of chemotherapy and time since treatment. This demonstrated no effect of diagnosis, chemotherapy regimen, number of cycles of chemotherapy or time since treatment on mood, sexual function or fatigue. In addition, the effect of treatment with XRT on fatigue scores and depression score did not reach statistical significance once it had been corrected for the other variables. Testosterone level significantly correlated with sexual activity ($P < 0.01$) and sexual interest ($P < 0.05$), whilst age inversely correlated with these two measures ($P < 0.05$). There were significant correlations between mood, sexual function and fatigue which are illustrated in Table 6.

### DISCUSSION

We have assessed several aspects of quality of life in men following treatment with cytotoxic chemotherapy, with or without radiotherapy. We have attempted to ascertain the contribution of mild Leydig cell dysfunction to alterations of sexual function, energy levels and mood by comparing a group of men with raised LH and low/normal testosterone levels with a group with normal hormone levels. Where possible, we have also compared the results with existing data from normal healthy men. Because of the large number of comparisons performed, the significance of some of the results must be interpreted with caution.

We found no difference in anxiety or depression score between patients with Leydig cell dysfunction and those with normal hormone levels. There is some evidence that overt hypogonadism is associated with alterations in mood and that these are reversed by androgen replacement (Wang et al, 1996). Our data suggest, however, that the milder degrees of testosterone deficiency in our cohort have no significant effect on mood. The rate of anxiety using the two suggested thresholds (8 and 11) was higher than that observed in normal healthy adults. Forty-one per cent and 15% of the patients had anxiety scores greater than seven and ten compared with 20% and 8% of a normal population studied by Lisspers et al 1997). It is not surprising, however, that higher rates of anxiety should be found in a group of men with a previous diagnosis of cancer, and results from other studies using the HADS carried out in patients treated for cancer have shown similar rates of anxiety (Moorey et al, 1991; Carroll et al, 1993; Loge et al, 1997).

The questionnaire regarding sexual function showed reduced sexual activity in the patients with Leydig cell dysfunction compared with those with normal hormone levels, and multivariate analysis demonstrated an effect of testosterone levels on sexual interest and activity. Several studies have found high rates of sexual dysfunction in treated cancer patients. Sexual problems were reported by 24% of treated Hodgkin's disease patients studied by Kornblith et al (1992) and less interest in sexual activity was reported by 20% of a similar group studied by Fobair et al (1986). Rates of sexual dysfunction following BMT have been described in between 20 and 50% of patients (Hjermstad and Kassa, 1995; Marks et al, 1997). Testosterone is important for determining sexual desire and erectile function and will therefore impact on sexual activity. Overt hypogonadism is associated with reduced sexual function which responds to androgen replacement (Arver et al, 1996), but the effect of milder degrees of testosterone deficiency have not been clearly defined. Whilst there are a number of possible factors contributing to the reduction in sexual function following cancer treatment (e.g. psychological stress associated with a life-threatening disorder or an effect of chemotherapy or XRT on penile blood flow or nerve supply), the difference in sexual activity that we observed between the groups suggests that mild Leydig cell insufficiency may be important.

We observed no difference in fatigue scores between patients in groups 1 and 2, suggesting that mild hypogonadism is not an important factor in determining energy levels in these patients. Fatigue scores in our subjects were, however, significantly greater than those observed in normal individuals. This is in contrast to results using the MFI-20 in patients who received radiotherapy for various malignancies, in whom no significant increase in any dimension of fatigue was demonstrated (Smets et al, 1998). We found significant correlations between mood and level of fatigue and there was a significantly higher level of general fatigue in those patients who received XRT. This suggests that treatment with XRT is aetiologically important in the development of fatigue in these patients and that mood may affect the perception of tiredness.

There are numerous studies which have reported high rates of fatigue during treatment of malignancies with XRT or chemotherapy (Smets et al, 1993). There are few published data, however, concerning the incidence of fatigue in long-term survivors of cancer, and most of the studies have not used questionnaires specifically designed to measure energy levels. Fatigue has been reported to occur commonly in survivors of Hodgkin's disease. Devlen et al (1987) reported loss of energy in 42% and tiredness in 32% of 120 patients with Hodgkin's disease and non-

© 2000 Cancer Research Campaign

Hodgkin's lymphoma 1 year after the diagnosis, despite the fact that the majority of patients had finished treatment and were in remission. A cross-sectional survey of long-term survivors of Hodgkin's disease a median of 9 years after treatment indicated that energy levels had not returned to patients' satisfaction in 37% (Fobair et al, 1986). In addition, in keeping with our results, they found an association between depression and energy levels in these patients (Fobair et al, 1986).

The mechanism by which cancer therapy causes fatigue in long-term survivors is not understood. Several possibilities exist to explain a reduction in energy during treatment, such as poor nutrition, the accumulation of toxic metabolites, anaemia or the side-effects of certain drugs. However, none of these adequately explain the persistence of fatigue long after the cessation of treatment. Abnormal mood states are more common in patients following cancer treatment and can be associated with decreased energy levels. We have been able to confirm that the level of depression correlates with fatigue in our cohort, although the lack of any significant increase in depression score compared with normal men suggests that this is not the only cause. In addition, our data confirm that treatment with radiotherapy is associated with increased fatigue in survivors of malignancy. We had postulated that mild Leydig cell dysfunction may be one mechanism by which cancer treatment may result in long-term fatigue. However, the lack of any significant difference in fatigue score between the patient groups in our study argues against an effect of mild hypogonadism.

In summary, we have shown higher levels of fatigue and anxiety in a group of patients who have been successfully treated with cytotoxic chemotherapy ± XRT for various malignancies compared with normal healthy men. Our data indicate that mild Leydig cell dysfunction is not the cause for these abnormalities, but may have an effect on sexual function. The results do not provide a convincing argument for mandatory androgen replacement therapy in the majority of these patients, although it may be beneficial in a minority. To establish whether androgen therapy is ever of any benefit in these patients a randomized placebo-controlled trial will be necessary.

### REFERENCES

Aass N, Fossa SD, Dahl AA and Moe TJ (1997) Prevalence of anxiety and depression in cancer patients seen at the Norwegian Radium Hospital. *Eur J Cancer* **33:** 1597–1604

Ahles TA, Tope DM, Furstenberg C, Hann D and Mills L (1996) Psychologic and neuropsychologic impact of autologous bone marrow transplantation. *J Clin Oncol* **14:** 1457–1462

Arver S, Dobs AS, Meikle AW, Allen RP, Sanders SW and Mazer NA (1996) Improvement of sexual function in testosterone deficient men treated for 1 year with a permeation enhanced testosterone transdermal system. *J Urol* **155:** 1604–1608

Berglund G, Bolund C, Fornander T, Rutqvist LE and Sjoden PO (1991) Late effects of adjuvant chemotherapy and postoperative radiotherapy on quality of life among breast cancer patients. *Eur J Cancer* **27:** 1075–1081

Bjordal K, Kaasa S and Mastekaasa A (1994) Quality of life in patients treated for head and neck cancer: a follow-up study 7 to 11 years after radiotherapy. *Int J Radiat Oncol Biol Phys* **28:** 847–856

Bloom JR, Fobair P, Gritz E, Wellisch D, Spiegel D, Varghese A and Hoppe R (1993) Psychosocial outcomes of cancer: a comparative analysis of Hodgkin's disease and testicular cancer. *J Clin Oncol* **11:** 979–988

Burris AS, Banks SM, Carter CS, Davidson JM and Sherins RJ (1992) A long-term, prospective study of the physiologic and behavioral effects of hormone replacement in untreated hypogonadal men. *J Androl* **13:** 297–304

Carroll BT, Kathol RG, Noyes R, Jr, Wald, TG and Clamon GH (1993) Screening for depression and anxiety in cancer patients using the Hospital Anxiety and Depression Scale. *Gen Hosp Psychiatry* **15:** 69–74

Devlen J, Maguire P, Phillips P and Crowther D (1987) Psychological problems associated with diagnosis and treatment of lymphomas. II: Prospective study. *Br Med J Clin Res Ed* **295:** 955–957

Fobair P, Hoppe RT, Bloom J, Cox R, Varghese A and Spiegel D (1986) Psychosocial problems among survivors of Hodgkin's disease. *J Clin Oncol* **4:** 805–814

Greenberg DB, Kornblith AB, Herndon JE, Zuckerman E, Schiffer CA, Weiss RB, Mayer RJ, Wolchok SM and Holland JC (1997) Quality of life for adult leukemia survivors treated on clinical trials of Cancer and Leukemia Group B during the period 1971–1988: predictors for later psychologic distress. *Cancer* **80:** 1936–1944

Hjermstad MJ and Kaasa S (1995) Quality of life in adult cancer patients treated with bone marrow transplantation – a review of the literature. *Eur J Cancer*, **31a:** 163–173

Howell SJ, Radford JA and Shalet SM (1999) Testicular function following cytotoxic chemotherapy – evidence of Leydig cell insufficiency. *Clin Oncol* (In press)

Ibbotson T, Maguire P, Selby P, Priestman T and Wallace L (1994) Screening for anxiety and depression in cancer patients: the effects of disease and treatment. *Eur J Cancer* **30a,** 37–40

Kornblith AB, Anderson J, Cella DF, Tross S, Zuckerman E, Cherin E, Henderson ES, Canellos GP, Kosty MP, Cooper MR, Weiss RB, Gottleib A and Holland JC (1992) Comparison of psychological adaptation and sexual function of survivors of advanced Hodgkin disease treated by MOPP, ABVD, or MOPP alternating with ABVD, *Cancer*, **70,** 2508–2516

Lisspers J, Nygren A, and Soderman E (1997) Hospital Anxiety and Depression Scale (HAD): some psychometric data for a Swedish sample. *Acta Psychiatry Scand*, **96,** 281–286

Loge JH, Abrahamsen AF, Ekeberg O, Hannisdal E and Kaasa S (1997) Psychological distress after cancer cure: a survey of 459 Hodgkin's disease survivors. *Br J Cancer*, **76,** 791–796

Marks S, Friedman SH, Delli Carpini L, Nezu CM and Nezu AM (1997) A prospective study of the effects of high-dose chemotherapy and bone marrow transplantation on sexual function in the first year after transplant. *Bone Marrow Transplant*, **19,** 819–822

Moorey S, Greer S, Watson M, Gorman C, Rowden L, Tunmore R, Robertson B and Bliss J (1991) The factor structure and factor stability of the hospital anxiety and depression scale in patients with cancer [see comments]. *Br J Psychiatry*, **158,** 255–259

Razavi D, Delvaux N, Farvaques C and Robaye E (1990) Screening for adjustment disorders and major depressive disorders in cancer in-patients [see comments]. *Br J Psychiatry*, **156,** 79–83

Smets EM, Garssen B, Schuster Uitterhoeve AL and de Haes JC (1993) Fatigue in cancer patients. *Br J Cancer*, **68,** 220–224

Smets EM, Garssen B, Cull A and de Haes, JC (1996) Application of the multidimensional fatigue inventory (MFI–20) in cancer patients receiving radiotherapy. *Br J Cancer*, **73,** 241–245

Smets EM, Visser MR, Willems Groot AF, Barssen B, Schuster Uitterhoeve AL and de Haes JC (1998) Fatigue and radiotherapy: (B) experience in patients 9 months following treatment. *Br J Cancer*, **78,** 907–912

Wang C, Alexander G, Berman N, Salehian B, Davidson T, McDonald V, Steiner B, Hull L, Callegari C and Swerdloff RS (1996) Testosterone replacement therapy improves mood in hypogonadal men: -a clinical research canter study. J *Clin Endocrinol Metab*, **81,** 3578–3583

Zigmond AS and Snaith RP (1983) The hospital anxiety and depression scale. *Acta Psychiatry Scand*, **67,** 361–370

*© 2000 Cancer Research Campaign*
